# Antioxidant effect of lycopene-enriched tomato paste on *N*-nitrosodiethylamine-induced oxidative stress in rats

**DOI:** 10.1007/s13105-014-0367-7

**Published:** 2014-11-13

**Authors:** Malgorzata Kujawska, Malgorzata Ewertowska, Teresa Adamska, Czeslaw Sadowski, Ewa Ignatowicz, Jadwiga Jodynis-Liebert

**Affiliations:** 1 Department of Toxicology, Poznan University of Medical Sciences, 30 Dojazd Str., 60-631 Poznań, Poland; 2 Department of Pharmaceutical Biochemistry, Poznan University of Medical Sciences, 4 Święcicki Str., 60-781 Poznań, Poland

**Keywords:** Lycopene, Antioxidant enzymes, Lipid peroxidation, Comet assay, Protein carbonyls

## Abstract

Lycopene is a carotenoid pigment produced by vegetables and fruits, with tomatoes and their processed products being the most abundant sources. A high number of conjugated dienes make lycopene a powerful radical scavenger. Its antioxidant properties are considered to be primarily involved in many beneficial health effects. The present study was designed to assess the protective effect of lycopene-enriched tomato paste against *N*-nitrosodiethylamine (NDEA)-induced oxidative stress in rats. Forty-eight male Wistar rats were divided randomly into six groups. Four groups were treated with tomato paste, per os, for 28 days in doses which were equivalent to 0.5 (groups II and V) and 2.5 mg/kg b.w./day of lycopene (groups III and VI). Rats from groups IV–VI were given intraperitoneally a single dose of NDEA, 150 mg/kg b.w. Group I (control) was given distilled water. Pretreatment with tomato paste protected the antioxidant enzymes: superoxide dismutase, catalase and glutathione reductase. Their activity was recovered by 32–97 %, as compared to NDEA-treated rats. Microsomal lipid peroxidation in the liver was decreased in rats pretreated with a lower dose of tomato paste by 28 %, as compared to animals given NDEA alone. Pretreatment with tomato paste caused a decrease in plasma concentration of protein carbonyls, even below the control level, in rats given NDEA. Moreover, a 10 % reduction of DNA damage in leucocytes caused by NDEA was observed. The tomato paste tested was able to suppress NDEA-induced oxidative stress in rats.

## Introduction

To optimise health status and to reduce the risk of oxidative stress-based diseases, a diet rich in natural antioxidants is recommended. Therefore, research work has been undertaken to uncover potential new sources of raw materials for use as functional food products [49].

Lycopene, a representative of carotenoids, is a red lipophilic pigment containing 11 conjugated double bonds. It is produced by vegetables and fruits, with tomatoes and processed tomato products being the most abundant sources [42, 44].

Epidemiological evidence suggests that consumption of lycopene is able to reduce the risk of chronic diseases such as cancer, cardiovascular diseases as well as psychiatric disorders and asthma [44]. Several mechanisms implicated in the health-beneficial effects of lycopene have been proposed. Because of a high number of conjugated dienes, lycopene is especially effective in scavenging the singlet oxygen and its ability is twice higher than that of β-carotene and ten times higher than that of α-tocopherol [32]. The antioxidant property of lycopene is considered to be primarily involved in its beneficial health effects. However, modulation of intracellular gap junction communication and hormonal, immune system, and metabolic pathways have been also reported [32]. Recently, it has been hypothesised that lycopene directly modulates several redox-sensitive signalling pathways being responsible for cell regulatory function, such as antioxidant response element (ARE), reactive oxygen species (ROS)-producing enzymes, small GTPases, mitogen-activated protein kinases (MAPK), nuclear factor κB (NF-κB), activator protein 1 (AP-1) as well as redox-sensitive proteins involved in modulation of cell cycle and apoptosis (p53, Bcl-2 family proteins and Ku protein) [32].

Lycopene is absorbed via passive diffusion and active process in which the scavenger receptor class B type 1 protein (SR-B1) transporter is engaged. The food matrix hinders efficient absorption of lycopene. Food processing and cooking release it from the food matrix and improve its intestinal absorption [50]. It has been reported that the amount of lycopene present in processed foods is often higher than in fresh vegetables; for example, ketchup contains 9.9–13.44 mg lycopene per 100 g wet weight, whereas fresh tomatoes contain 0.88–7.44 mg lycopene per 100 g wet weight [44].

The World Cancer Research Fund Report 2007 estimates that most of the cancer deaths are attributed to lifestyle factors and the majority of them are linked to environmental exposures [51]. *N*-Nitrosamines are a group of environmental chemicals known to be metabolised to carcinogenic and pro-oxidant agents. They are formed endogenously in the acidic condition of the stomach from nitrites and/or amines. Amines are commonly found in a wide variety of food constituents, residues of agricultural chemicals or pharmaceutical drugs. In turn, nitrites are formed by the reduction of ingested nitrates present in cured meat, beer, cheese, sausage, smoked fish or dried milk. Additionally, intestinal bacteria are able to reduce nitro-substituted compounds using the reducing power of NAD(P)H [1, 25]. A representative of this group, *N*-nitrosodiethylamine (NDEA), generates carbocations and ROS during cytochrome P450-mediated biotransformation [48]. Liver injury induced by NDEA is a well-known model of hepatotoxicity commonly used for the screening of the hepatoprotective activity of natural compounds [22–24, 33].

The present study was designed to evaluate the efficiency of lycopene-enriched tomato paste in alleviating NDEA-induced oxidative stress in rats.

## Materials and methods

### Materials

Tomato paste used in this study was made by Cinna Health Products, Poland. To produce tomato paste, tomato concentrate was diluted in acidic water, and the mixture was centrifuged by continuous-flow centrifugation at 6000*g*. The tomato paste product contained 3 mg of lycopene/g of paste.

### Experimental design

Forty-eight male Wistar rats (250 ± 15 g) bred in the Department of Toxicology, Poznan University of Medical Sciences, were used in the experiment. The rats were housed in an animal facility at 22 ± 1 °C, 50 % humidity and controlled circulation of air with a 12-h light/dark cycle and fed certified laboratory feed (Labofeed H, ISO 22000). Food and drinking water were available ad libitum.

The rats were divided randomly into six groups, eight animals in each, as shown in Table 1. Groups II and V were given by gavage tomato paste suspended in 2 ml of water in the dose 165 mg/kg b.w./day, which was equivalent to the dose of lycopene 0.5 mg/kg b.w./day. Groups III and VI were administered tomato paste in the dose 825 mg/kg b.w./day (the equivalent of 2.5 mg/kg b.w./day of lycopene). The tomato paste treatment was continued for 28 days. Groups I (control) and IV were given every day 2 ml of distilled water. On the 27th day of the experiment, rats from groups IV–VI were given intraperitoneally a single dose of NDEA, 150 mg/kg b.w. After 24 h, the rats were anaesthetised with ketamine-xylazine, blood was withdrawn from the heart and the liver was excised. A portion of whole heparinised blood was separated for the comet assay; the remaining blood was centrifuged (3000 rpm, +4 °C) for plasma separation. Immediately after removal, the livers were perfused with ice-cold 1.15 % KCl. One portion was homogenised in buffered Tris/sucrose solution (pH 7.55) for microsomal and cytosol fractions preparation, and the rest of the tissue was stored at −80 °C for glutathione determination. Liver homogenate for glutathione determination was prepared in a phosphate buffer, pH 7.4. Subcellular fractions were prepared by differential centrifugation according to the standard procedure. In each fraction, a protein concentration was determined using Folin-Ciocalteu reagent.

**Table 1:** Treatment protocol

Group	Treatment
I	Controls
	Distilled water (p.o.)
II	Tomato paste I
	0.5 mg lycopene/kg b.w./day (p.o.)
III	Tomato paste II
	2.5 mg lycopene/kg b.w./day (p.o.)
IV	*N*-Nitrosodiethylamine (NDEA)
	150 mg/kg b.w. (i.p.)
V	Tomato paste I (0.5 mg lycopene/kg b.w./day, p.o.) + NDEA (150 mg/kg b.w., i.p.)
VI	Tomato paste II (2.5 mg lycopene/kg b.w./day, p.o.) + NDEA (150 mg/kg b.w., i.p.)

The experiment was performed according to the Local Animal Ethics Committee guidelines for animal experimentation.

### Biochemical assays

Lipid peroxidation in the liver was assayed both in native microsomal fraction and after stimulation with Fe^2+^/ascorbate. The level of microsomal lipid peroxidation was assessed by measuring thiobarbituric acid reactive substances (TBARS) [39].

Reduced glutathione in the liver was determined by its reaction with Ellman’s reagent [40].

Antioxidant enzymes were assayed in the liver cytosol. Superoxide dismutase (SOD) activity was determined on the basis of the inhibition of spontaneous epinephrine oxidation [19]. Catalase (CAT) activity was assayed by the measurement of the rate of H_2_O_2_ decomposition [19].

Glutathione peroxidase (GPx) activity was determined by the method of Mohandas et al. [28] using hydrogen peroxide as a substrate. The rate of the NADPH disappearance at 340 nm was a measure of the enzyme activity.

Glutathione reductase (GR) activity was assayed by measuring NADPH oxidation at 340 nm in the presence of oxidised glutathione [28].

Protein carbonyl concentration in plasma was determined using a commercial ELISA assay kit from BioCell Corporation, NZ (BioCell), based on the method described by Buss et al. [7].

A comet assay in alkaline condition (pH > 13) was conducted in the whole blood leucocytes according to the method of Hartmann et al. [16]. Heparinized blood was processed immediately after a collection from the heart. Blood samples embedded in low-melting-point agarose were submitted to cell lysis, DNA unwinding, electrophoresis and neutralisation and then were dehydrated in absolute ethanol, dried, stored at room temperature and protected from light. Before microscopic evaluation, the slides were rehydrated and stained with ethidium bromide. Two slides were made for each blood sample. Images of nucleoids from a Zeiss fluorescence microscope (magnification ×400) were captured with a digital camera. One hundred nucleoids were scored in each slide. The nucleoids were divided into five groups according to the degree of DNA damage and graded from 0 (no damage) to 4 (maximal damage) [9]. A total damage score for the slide was derived by multiplying the number of cells assigned to each grade of damage by the numeric value of the grade and summing over all grades.

### Statistical analysis

The data were expressed as mean ± SD. One-way analysis of variance (ANOVA) followed by the Tukey-Kramer multiple comparisons test was used. *p* < 0.05 was considered to be the limit of significance.

## Results

NDEA exposure caused an increase in hepatic microsomal lipid peroxidation by 109 % in the uninduced assay and by 60 % in the Fe^2+^/ascorbate-stimulated assay, as compared to the control group (Table 2). Pretreatment with the lower dose of tomato paste (165 mg/kg b.w./day) appeared to be more efficient in attenuating oxidative stress in rats receiving NDEA. In this group, the TBARS level in native microsomes was significantly lower, by 28 %, than that in animals given NDEA alone. However, the same dose of tomato paste alone caused an increase in the level of uninduced lipid peroxidation, by 37 %. Fe^2+^/ascorbate-stimulated lipid peroxidation was attenuated by both doses of tomato paste pretreatment by 35 and 33 %, respectively.

**Table 2:** Effect of tomato paste pretreatment on microsomal lipid peroxidation and reduced glutathione in the liver of rats given *N*-nitrosodiethylamine (NDEA)

Treatment	Microsomal lipid peroxidation (nmol TBARS /min/mg protein)		GSH (μmol/g tissue)
	Uninduced	Fe^2+^/ascorbate	
Controls	0.54 ± 0.07	13.6 ± 1.9	6.0 ± 0.6
Tomato paste I 0.5 mg lycopene/kg b.w./day	0.62 ± 0.14 [↑14 %]	14.3 ± 2.8	6.1 ± 0.6
Tomato paste II 2.5 mg lycopene/kg b.w./day	0.74 ± 0.11 [↑37 %]^a^	15.2 ± 2.3	6.4 ± 0.7
*N*-Nitrosodiethylamine (NDEA) 150 mg/kg b.w.	1.13 ± 0.22 [↑109 %]^a^	21.8 ± 1.5 [↑60 %]^a^	10.3 ± 1.3 [↑73 %]^a^
Tomato paste I (0.5 mg lycopene/kg b.w./day) + NDEA (150 mg/kg b.w.)	0.81 ± 0.12 [↓28 %]^b^	14.0 ± 2.2 [↓35 %]^b^	10.3 ± 1.7
Tomato paste II (2.5 mg lycopene/kg b.w./day) + NDEA (150 mg/kg b.w.)	0.92 ± 0.23 [↓19 %]	14.6 ± 2.2 [↓33 %]^b^	11.4 ± 0.9

Results are mean ± SD, *n* = 8. Control rats were given water. Values in brackets express percent of change

^a^Controls are compared with tomato paste only or NDEA-only-treated groups. *p* < 0.05

^b^The NDEA-treated group is compared with the tomato paste + NDEA-treated groups. *p* < 0.05

NDEA alone caused a significant increase in hepatic glutathione level, by 73 %, as compared to the controls. Tomato paste pretreatment did not affect an elevated glutathione (GSH) concentration in NDEA-treated animals. Tomato paste alone did not change the basal GSH concentration either (Table 2).

All investigated antioxidant enzymes were inhibited in animals treated with NDEA alone by 16–73 %, as compared to controls (Table 3). Pretreatment with the lower dose of tomato paste caused a recovery of catalase (CAT) and glutathione reductase (GR) activity by 74 and 97 %, respectively, and slight insignificant increase (by 17 %) in glutathione peroxidase (GPx) activity. Administration of the higher dose of tomato paste also produced a rise in CAT and GR activity by 51 and 32 %, respectively, and additionally in superoxide dismutase (SOD) activity by 32 %, as compared to NDEA-treated rats. Tomato paste alone caused GPx activity induction approximately by 30 %. In contrast, activities of SOD and CAT in the same groups of rats were slightly decreased by 15–33 %, as compared to control rats. GR was the only enzyme not affected significantly by tomato paste alone (Table 3).

**Table 3:** Effect of tomato paste pretreatment on hepatic antioxidant enzymes in rats given *N*-nitrosodiethylamine (NDEA)

Treatment	SOD (U/mg)	CAT (U/mg)	GPx (nmol NADPH/min/mg protein)	GR (nmol NADPH/min/mg protein)
Controls	4.09 ± 0.45	4.05 ± 0.46	514.2 ± 60.3	82.2 ± 10.6
Tomato paste I 0.5 mg lycopene/kg b.w./day	3.41 ± 0.36 [↓17 %]^a^	3.43 ± 0.46 [↓15 %]^a^	704.2 ± 60.16 [↑37 %]^a^	95.0 ± 5.3 [↑16 %]
Tomato paste II 2.5 mg lycopene/kg b.w./day	3.36 ± 0.48 [↓18 %]^a^	2.71 ± 0.44 [↓33 %]^a^	670.6 ± 63.4 [↑30 %]^a^	66.5 ± 15.2 [↓19 %]
*N*-Nitrosodiethylamine (NDEA) 150 mg/kg b.w.	2.18 ± 0.19 [↓47 %]^a^	1.08 ± 0.36 [↓73 %]^a^	430.7 ± 25.8 [↓16 %]^a^	32.6 ± 4.3 [↓60 %]^a^
Tomato paste I 0.5 mg lycopene/kg b.w./day + NDEA (150 mg/kg b.w.)	2.15 ± 0.55	1.87 ± 0.23 [↑74 %]^b^	505.6 ± 36.4 [↑17 %]	64.1 ± 11.2 [↑97 %]^b^
Tomato paste II 2.5 mg lycopene/kg b.w./day + NDEA (150 mg/kg b.w.)	2.88 ± 0.33 [↑32 %]^b^	1.63 ± 0.32 [↑51 %]^b^	468.5 ± 53.4 [↑9 %]	43.0 ± 8.8 [↑32 %]^b^

Results are mean ± SD, *n* = 8. Control rats were given water

Values in brackets express percent of change

^a^Controls are compared with tomato paste only or NDEA-only-treated groups. *p* < 0.05

^b^The NDEA-treated group is compared with the tomato paste + NDEA-treated groups. *p* < 0.05

Protein carbonyl (PC) concentration in plasma of rats treated with NDEA alone was higher by 24 %, as compared to the control group (Table 4). Pretreatment with the lower dose of tomato paste was found to protect plasma proteins against oxidative damage caused by NDEA, and a significant decrease in the PC content, even below the control level, was observed. No significant effect of either of the administered doses of tomato paste on PC concentration was noticed, when applied alone.

**Table 4:** Effect of tomato paste pretreatment on protein carbonyl concentration in the plasma and the DNA damage in blood leucocytes of rats given *N*-nitrosodiethylamine (NDEA)

Treatment	PCs (nmol/mg protein)	DNA damage (arbitrary points)
Controls	0.54 ± 0.05	73.3 ± 7.3
Tomato paste I 0.5 mg lycopene/kg b.w./day	0.50 ± 0.07 [↓9 %]	69.8 ± 10.4
Tomato paste II 2.5 mg lycopene/kg b.w./day	0.48 ± 0.11 [↓12 %]	77.5 ± 9.3
*N*-Nitrosodiethylamine (NDEA) 150 mg/kg b.w.	0.68 ± 0.13 [↑24 %]^a^	131.5 ± 7.5 [↑80 %]^a^
Tomato paste I (0.5 mg lycopene/kg b.w./day) + NDEA (150 mg/kg b.w.)	0.41 ± 0.07 [↓40 %]^b^	127.4 ± 3.9
Tomato paste II (2.5 mg lycopene/kg b.w./day) + NDEA (150 mg/kg b.w.)	0.61 ± 0.10 [↓10 %]	118.3 ± 5.1 [↓10 %]^b^

Results are mean ± SD, *n* = 8. Control rats were given water. Values in brackets express percent of change

^a^Controls are compared with tomato paste only or NDEA-only-treated groups. *p* < 0.05

^b^The NDEA-treated group is compared with the tomato paste + NDEA-treated groups. *p* < 0.05

**Fig. 1:**
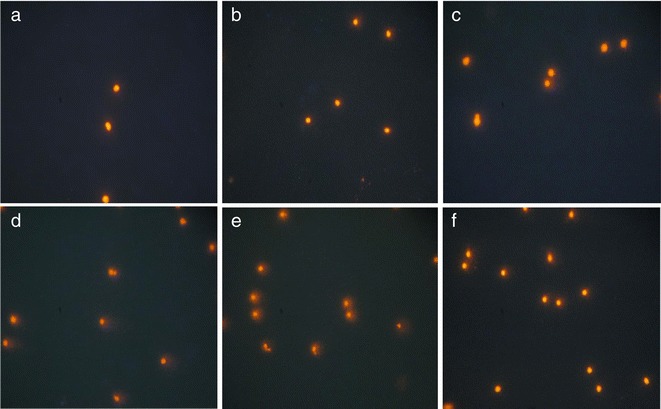
Fluorescence microscope-derived pictures of DNA damage in blood leucocytes of **a** control rats, **b** rats given tomato paste I, **c** rats given tomato paste II, **d** rats given *N*-nitrosodiethylamine (NDEA), **e** rats given tomato paste I + NDEA and **f** rats given tomato paste II + NDEA measured by alkaline comet assay. For each sample, 100 nucleoids in two separate slides were scored

DNA damage measured in the whole blood leucocytes was also increased significantly, by 80 %, in the NDEA-alone-treated group. Only the higher dose of tomato paste caused a slight (by 10 %) but statistically significant reduction of DNA damage, in comparison to animals receiving NDEA alone (Table 4 and Fig. 1).

## Discussion

An increasing number of reports have shown that lycopene may decrease the risk of many chronic diseases, in particular cancer. Several studies have established the strong relationship between these preventative effects and its antioxidant activity [32, 44].

As NDEA was proved to induce oxidative damage to cellular macromolecules [52, 53], we have used it in our experimental protocol as a model pro-oxidant. The same experimental design has been used in our previous studies for the evaluation of potential antioxidant activity of fruit juices [22–24].

One of the markers of oxidative stress is the level of lipid peroxidation products which are formed from polyunsaturated fatty acids during a free radical chain reaction in cellular membranes [30]. In our experiment, pretreatment with tomato paste significantly suppressed NDEA-induced elevation of the level of lipid peroxidation both in native and stimulated with Fe^2+^/ascorbate microsomes. Similar results were reported by Dogukan et al. [13], who treated with lycopene (for 10 days) rats administered concomitantly cisplatin. As a result, about 40 % reduction in lipid peroxidation level in the kidney was observed.

It is unclear why the higher dose of tomato paste alone increased the level of an uninduced lipid peroxidation. However, Jomova et al. [20] reported that lycopene at the concentration of 30 μg/g in hexane acts as a pro-oxidant in a system consisting of oxidised triglycerides. An increase in the amount of hydroperoxides formed in the triglyceride system containing lycopene as compared to control (no lycopene added) was observed [20]. Such a paradoxical effect was also reported for another class of antioxidants, namely polyphenols [6, 14].

The major constituents of the antioxidant defence system in cells are antioxidant enzymes. They play a significant role in protecting cellular systems from oxidative damage induced by xenobiotics, and thus, measurement of their activity is used to assess the redox status. Depending on the intensity and duration of the stress as well as susceptibility of exposed living organisms, both induction and inhibition of antioxidant enzymes are observed. It has been revealed that if the amount of ROS reaches the *threshold level* which overwhelms the antioxidant capacities, irreversible damage will occur [45]. Similar to the results from our previous studies [22–24], activities of antioxidant enzymes were markedly inhibited in the liver of NDEA-treated animals. In line with our observations, Pradeep et al. [34] also reported that a single intraperitoneal administration of NDEA (200 mg/kg) to rats resulted in a significant decrease in the activity of antioxidant enzymes. This decrease with a concomitant increase in lipid peroxidation level in the liver could be attributed to their inactivation by free radicals generated in the process of NDEA biotransformation [34].

In the present study, the treatment with tomato paste caused a recovery of the decreased activity of antioxidant enzymes. Superoxide dismutase (SOD) is the primary superoxide scavenger and has become an important potential chemopreventive modality in view of the link between ROS and carcinogenesis [45]. In the present study, only the higher dose of tomato paste caused an increase in SOD activity in NDEA-treated rats.

The other enzyme considered as the first line of defence against ROS is catalase (CAT). Both these enzymes cooperate together; catalase decomposes H_2_O_2_ formed during the SOD-catalysed dismutation reaction of superoxide radical anion (O^2−^) [45]. Both doses of tomato paste abrogated NDEA-induced inhibition in CAT activity. Atessahin et al. [4] reported that lycopene pre- and post-treatment restored cisplatin-induced impairment in CAT activity in the rat kidney. Administration of lycopene at 100, 200 and 300 mg/kg to the rats with *N*-methyl-*N*′-nitro-*N*-nitrosoguanidine-induced oxidative injury also significantly restored both blood and gastric activities of the antioxidant enzymes: SOD, CAT and GPx [26]. On the other hand, in the present study administration of tomato paste alone caused a decrease in both hepatic SOD and CAT activities in rats. These results are in concurrence with findings reported by Moreira et al. [29] where the rats treated with tomato powder (corresponding to approximately 20–30 mg lycopene/kg/day) for 28 days demonstrated reduced activity of hepatic SOD and CAT, as compared to control rats. However, Hu et al. [17] observed a significant increase in SOD and CAT activity in the livers of mice administered high doses of *Blakeslea trispora* powder corresponding to 534 and 1068 mg/kg b.w./day of lycopene.

Lycopene has been reported as a strong singlet oxygen quencher and shown to participate in the first line of antioxidant defence [31]. In light of these findings, it can be suggested that in normal rats, a long-term administration of lycopene may decrease SOD and CAT activity as a result of an adjustment of cell redox status to the exogenous antioxidant intake.

A recent study [10] revealed that the dietary and endogenous antioxidants interact together and that complex gene-diet interactions may further impact an individual’s ability to manage oxidative stress. On the other hand, it has been reported that antioxidants at high doses could also act as pro-oxidants and disrupt the redox balance [6]. Therefore, high doses of lycopene can cause decrease in SOD and CAT activity. On the basis of available data on the effect of lycopene on hepatic SOD and CAT activity, it could be suggested that a dose is a substantial factor affecting the alterations of these enzymes activity.

One of the remarkable effects of NDEA treatment on the antioxidant status of rat liver is a decrease in the activities of GSH-related enzymes. GPx catalyses the reduction of lipid peroxides and also H_2_O_2_ at the expense of GSH [17]. Reduced GPx activity causes accumulation of lipid peroxides and other oxidants which make the cellular membranes more susceptible to the oxidative damage [31]. In the current study, only a slight reduction in the activity of hepatic GPx in NDEA-treated rats was observed which was not attributed to the depletion of GSH but rather might be a consequence of covalent modification of a protein caused by free radicals generated during the NDEA metabolism. Reduced GPx activity was slightly restored by the pretreatment with the lower dose of tomato paste. Tomato paste alone caused an increase in hepatic GPx activity when compared to control rats. Similarly, a 30-day administration of *B. trispora* powder at a dose corresponding to 534 and 1068 mg lycopene/kg b.w. increased significantly the activity of hepatic GPx in adult mice [17]. Results from various laboratories have shown that administration of 1068 mg lycopene/kg b.w. for 30 days [17] or 10 mg/kg for 21 days [3] led to an increase in GPx activity also in the kidney of rodents. As the transcription factor Nrf2 plays a key role in the antioxidant response element (ARE)-mediated expression of many antioxidant and phase 2 detoxifying enzymes, possible involvement of Nrf2 in the observed increase in GPx activity might be suggested. However, there is no available information about the mechanisms involved in the upregulation of antioxidant enzymes by lycopene [27].

The activity of GPx is coupled to glutathione reductase (GR), which regenerates the reduced form of GSH from glutathione disulphide GSSG [12]. Our data show that the lower dose of tomato paste completely prevented NDEA-induced inhibition of GR activity; the effect of the higher dose was less pronounced. These results are supported by several studies showing that preparations containing lycopene prevented inhibition of hepatic GR in rodents fed with a hyperenergetic [29] and high-cholesterol diet eliciting oxidative stress [8, 38].

Earlier studies from our laboratory [22–24] and the present results showed that 24 h after a single dose of NDEA (200 mg/kg b.w.), the increase in hepatic GSH concentration occurs, reflecting a possible adaptation mechanism. There are some discrepancies in reports concerning the effects of NDEA on GSH concentration in laboratory animals. Some authors reported depletion of GSH [5, 34, 37, 43], and others demonstrated an increase in GSH content [2, 18, 35, 36]. The reason for these discrepancies could be related to different protocols of the experiments including different periods between the administration of NDEA and collecting liver samples. Taking together all the available data, it can be suggested that a dose, application routes and regimes of dosing are crucial factors modulating the GSH biosynthesis. It was hypothesised that NDEA causes GSH depletion soon after administration, but after a phase of GSH deficiency, the level of GSH increases which is regarded as a hypercompensation mechanism [23]. Tomato paste did not affect the NDEA-induced increase in the hepatic glutathione level.

Oxidative stress markers were examined not only in the liver but also in blood, namely content of protein carbonyl groups (PCs); the level of DNA damage in blood leucocytes was also assayed. These two markers of oxidative damage in blood can be measured noninvasively, and hence, they may play a significant role in human nutrition intervention studies.

PCs are the ubiquitously used markers of protein oxidation. Moreover, PCs are more stable and persist in circulation for a longer period after exposure, as compared to lipid peroxidation products or other products of protein oxidation [41], and they are regarded to be sensitive to antioxidant treatment [11]. In our study, similarly to earlier findings [22, 24], we observed an increased concentration of protein carbonyls in plasma of NDEA-administered rats, reflecting oxidative protein damage. Pretreatment with a lower dose of tomato paste preserved NDEA-induced protein carbonyl formation; however, the higher dose was not efficient. In view of these observations, the ability of tomato paste to induce a compensatory response upon oxidative or electrophilic stress may depend on the dose. In this study, there is a consistency in the set of presented results concerning plasma protein carbonyls as well as hepatic lipid peroxidation and hepatic antioxidant enzymes (except SOD activity), namely the lower dose appeared to be more efficient in attenuating oxidative stress in rats receiving NDEA. So far, the explanation of these effects is not quite clear. Nonetheless, it could be partially elucidated on the basis of the report of Veeramachaneni et al. [47], who demonstrated that lycopene at a high dose (3.3 mg/kg b.w./day) enhanced the expression of hepatic CYP2E1 induced by ethanol in rats. Similarly, the pretreatment with the higher dose of tomato paste in our study might cause the activation of CYP2E1 that resulted in an enhanced bioactivation of NDEA as compared to animals given NDEA alone. It is generally known that biotransformation of NDEA mediated by CYP2E1 gives rise to the generation of ROS, leading to oxidative stress in rats [48]. It could be suggested that this process was probably responsible for unexpected weak antioxidant effects of the higher dose of tomato paste in NDEA-administered rats. Therefore, further research on the possible interaction between high doses of lycopene and chemical inducers of CYP2E1 is desirable.

As NDEA generates during its metabolism DNA-binding ethylcarbonium ions and reactive oxygen species, resulting in DNA lesions, an advisable approach from the chemopreventive standpoint involves the measurement of the level of DNA damage. The comet assay is a sensitive technique evaluating in quantitative way DNA damage. Results from various laboratories have shown that NDEA induced DNA damage as indicated by comet-shaped nuclei cells both in the liver [15, 21] and in blood leucocytes [22] of rats.

The observed increase in DNA damage in the whole blood leucocytes was possibly associated with the passing of reactive NDEA metabolites from the liver to the circulation and/or enhanced oxidative metabolism of stimulated neutrophils and macrophages. Ueno et al. [46] suggested that NDEA-derived ROS induce release of pro-inflammatory cytokines and nitric oxide from neutrophils activated via NF-κB-dependent pathways and led to oxidative damage of macromolecules. Thereby, it could be hypothesised that the suppression of the pro-inflammatory action of neutrophils caused by lycopene was responsible for the observed slight attenuation of DNA damage in the present study. It is noteworthy, however, that in another study the lycopene administration to NDEA-challenged mice caused a further increase in DNA damage in the liver when compared to the NDEA-treated group [15].

So far, no study was reported on the effect of lycopene on protein carbonyl concentration and the level of comet-shaped cells in the blood of animals affected by chemically induced oxidative stress.

In conclusion, the results of our present study clearly demonstrate that tomato paste enriched with lycopene is able to suppress NDEA-induced oxidative stress in rats. As no distinct relationship between dose and effect for various parameters was observed, further studies should be undertaken to elucidate this phenomenon.
